# Leaf-Shape Variation Affects the Validity of the Square-Law Relationship Between Leaf Area and Leaf Length: Evidence from 12 *Malus* Cultivars

**DOI:** 10.3390/plants15213359

**Published:** 2026-10-29

**Authors:** Wen Gu, Yimin Zhu, Zhongqin Li, Danyu Zhou, Yanbo Zhang, Wangxiang Zhang, Feixue Jiang, Dirk Hölscher, Peijian Shi

**Affiliations:** 1 College of Forestry and Grassland, Nanjing Forestry University, #159 Longpan Road, Nanjing 210037, China; gw0815@njfu.edu.cn (W.G.); zhongqin@njfu.edu.cn (Z.L.); zhoudy@njfu.edu.cn (D.Z.); malus2011@163.com (W.Z.); fxjiang@njfu.edu.cn (F.J.); 2 School of Forestry Science and Technology, Lishui Vocational and Technical College, #357 Xinyuan Street, Lishui 323000, China; yiminzhu@lszjy.edu.cn; 3 Lishui Forest Resources Protection and Management Station, #158 Kuocang Road, Lishui 323000, China; zhangyanbo6@126.com; 4 Tropical Silviculture and Forest Ecology, University of Göttingen, #1 Büsgenweg, 37077 Göttingen, Germany

**Keywords:** individual leaf area, leaf, *Malus*, Montgomery equation, square-law equation

## Abstract

The Montgomery equation (ME), *A* = *kLW*, provides a simple and non-destructive approach for estimating leaf area (*A*) from leaf length (*L*) and width (*W*), whereas the square-law equation (SLE) assumes that *A*∝*L*^2^. This study evaluated and compared the goodness of fit of the ME and SLE across 12 *Malus* cultivars and examined whether variation in leaf shape was associated with SLE performance. A total of 3679 leaves were analyzed, and the coefficient of variation in the ratio of leaf width to length was used to quantify within-cultivar leaf-shape variation. The ME provided a good fit for all 12 cultivars, with root-mean-square error (RMSE) values not exceeding 0.0285. For the pooled dataset, the estimated Montgomery parameter was 0.6641, with a 95% confidence interval of 0.6632–0.6650, and fell within the theoretical range of 1/2 to π/4. The ME consistently showed lower RMSE than the SLE, with the relative reduction in RMSE exceeding 49% for all cultivars. In addition, variation in the ratio of width to length was strongly positively correlated with the RMSE of the SLE. These findings suggest that variation in leaf shape is closely associated with the goodness of fit of the SLE and support the use of both leaf dimensions for more reliable leaf-area estimation. This study provides a practical basis for rapid and non-destructive estimation of leaf area in *Malus*.

## 1. Introduction

Leaf area is a key functional trait that directly reflects photosynthetic capacity, transpiration rates, and nutrient use efficiency, linking individual plant carbon gain to broader ecological strategies [1,2,3]. It not only directly determines the photosynthetic capacity at the individual leaf level but also influences canopy light distribution, transpirational cooling, and the plant’s carbon–water balance, thereby influencing growth dynamics and competitive ability [4]. Under global climate change, leaf size traits, especially leaf area, have been widely used as key indicators of plant adaptation to contrasting habitats [5,6,7]. For example, large leaves enhance light interception in the low-light understory of tropical rainforests, whereas small leaves reduce boundary layer thickness and overheating risk in arid or high-radiation environments [8,9]. Consequently, the development of rapid, accurate, and non-destructive methods for estimating leaf area is not only a fundamental requirement in plant ecophysiological studies but also provides essential technical support for understanding plant adaptive strategies under climate change.

Among the various methods for estimating leaf area (*A*), mathematical models based on leaf linear dimensions have attracted considerable attention because of their simplicity, non-destructiveness, and repeatability. First of all, the power-law equation (PLE) offers a flexible method for exploring scaling relationships between size, morphology, or physiological traits [10,11,12,13,14,15], including the relationships between *A* and *LW*. Moreover, Montgomery [16] proposed that *A* is proportional to the product of leaf length (*L*) and width (*W*) in estimating corn leaf area, i.e., *A* = *kLW*, where *k* is a constant. This isometry between *A* and *LW* is referred to as the Montgomery equation (ME) hereinafter. Prior studies have shown that the ME is applicable not only to simple-leaved species such as grasses and willows [17,18,19] but also to many other dicotyledonous species with elliptical, lanceolate, cordate, or even shallowly lobed leaves [20,21,22,23]. The ME has also been confirmed across diverse plant groups, including woody plants, herbs, and vines [20,21,23,24,25]. For example, Shi et al. [19] validated the ME for the combined data of 101 bamboo taxa with > 10,000 leaves; Ma et al. [26] demonstrated that the validity of ME is independent of tree size using > 6500 leaves from 60 individual trees of the alpine oak (*Quercus pannosa*). Importantly, the proportionality coefficient of the ME (i.e., *k*, denoted as the Montgomery parameter) generally ranges between 1/2 and π/4, corresponding to different leaf shapes, a geometric limit that reflects the evolutionary trade-off in the filling efficiency of a rectangle or an ellipse [25]. A simpler relationship, proposed by Thompson [12], assumes that *A* ∝ *L*^2^ and is referred to as the square-law equation (SLE). Because model performance can depend on how relevant dimensions and scales are represented [27], omitting leaf width may affect the goodness of fit of leaf-area relationships when leaf shape varies [20]. Although the ME has been evaluated across different species [20], its goodness of fit when data from multiple cultivars within the same genus are pooled remains unclear. Leaf-shape variation among cultivars of the same genus may be smaller than that among different species, potentially favoring a common relationship between *A* and *LW*. However, whether the ME maintains a good fit across pooled cultivar data has not been sufficiently examined. Previous studies have also suggested an association between variation in the ratio of leaf width to length (RWL) and the goodness of fit of the SLE [20,21]. Although RWL provides a useful measure of leaf-shape variation among closely related taxa [19,28], whether within-cultivar variation in RWL is similarly associated with the goodness of fit of the SLE across cultivars of the same genus remains unclear.

Crabapple (*Malus* spp.), a member of the Rosaceae family, is widely used in ornamental horticulture because of its colorful and abundant flowers and fruits [29,30]. This genus was selected because intensive breeding has resulted in cultivars exhibiting considerable variation in floral, leaf, and fruit characteristics [31,32,33]. However, no previous study has compared multiple *Malus* cultivars using large sample sizes to evaluate the applicability of ME and to examine how leaf-shape variation affects the performance of the SLE. Therefore, we analyzed 3679 leaves from 12 *Malus* cultivars, with more than 300 leaves per cultivar. We evaluated the ME and SLE at the individual cultivar level and for the combination of the 12 cultivars. We also estimated the Montgomery parameter for each cultivar and tested whether variation in the RWL was associated with the goodness of fit of the SLE. By comparing the performance of the two equations across cultivars with different leaf morphologies, this study also provides a practical basis for selecting a simple and reliable method for rapid, non-destructive leaf-area estimation in *Malus*. Such an approach can facilitate repeated measurements and large-scale phenotyping in horticultural, physiological, and ecological studies.

## 2. Materials and Methods

### 2.1. Leaf Sampling

**Figure 1:**
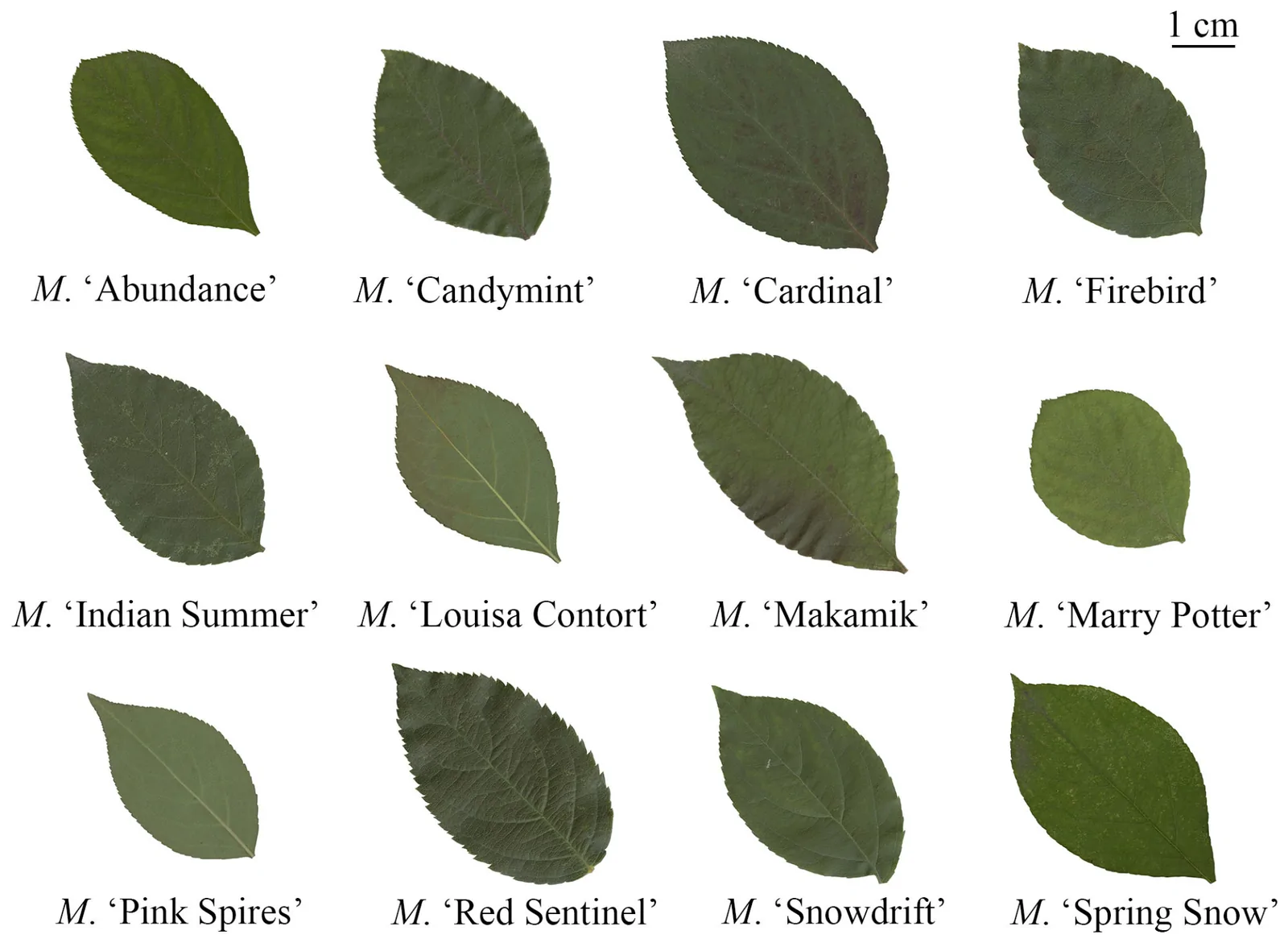
Representative leaves of the 12 *Malus* cultivars.

A total of 3679 fresh leaves of 12 *Malus* cultivars (shown in Figure 1) were sampled from the National Repository of Crabapple Germplasm in Yangzhou, Jiangsu Province, China (32°26′24″ N, 119°39′00″ E) from May to June 2025. For each cultivar, branches were randomly collected from at least 15 individual trees and from different crown orientations. Mature, fully expanded, and intact leaves with mature coloration and texture and no visible signs of ongoing expansion or senescence were then randomly selected from the collected branches until more than 300 leaves had been obtained for each cultivar; young, tender leaves near the shoot apex were excluded. The sampled leaves were placed in insulated boxes with ice packs and transported to the laboratory within two hours to prevent tissue dehydration.

### 2.2. Data Acquisition

**Figure 2:**
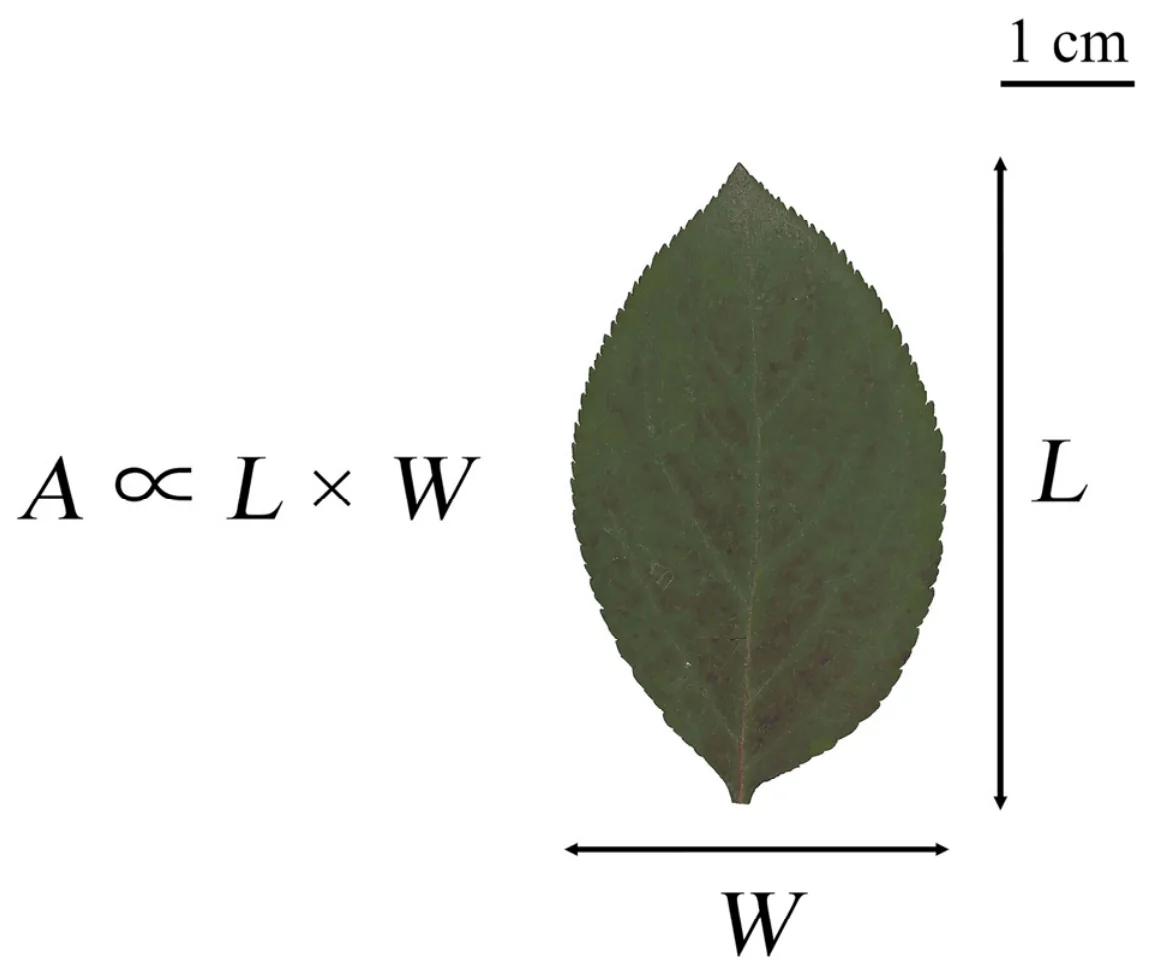
Illustration for the Montgomery equation (ME) and the corresponding definitions for the variables in the equation. *A* represents individual leaf area; *L* and *W* represent the length and width of the leaf, respectively.

Leaves were scanned at 600 dpi resolution and saved as JPEG (.jpg) images using a photo scanner (Epson V600, Batam, Indonesia). The leaf blades (excluding petioles) were isolated and then converted into black-and-white bitmap (.bmp) images using Adobe Photoshop 2021 (v22.4.2; Adobe Systems Incorporated, San Jose, CA, USA). The planar coordinates for each leaf profile were extracted using a MATLAB procedure (MATLAB ≥ R2009a; MathWorks, Natick, MA, USA) developed by Shi et al. [34] and Su et al. [35]. The “bilat” function in the “biogeom” package (version 1.5.1) [36] under R software (version 4.3.1) [37] was used to measure leaf area (*A* in cm^2^), length (*L* in cm, defined as the distance from the leaf apex to base), and width (*W* in cm, defined as the maximum width on the straight line perpendicular to the leaf length axis; Figure 2).

### 2.3. Statistical Analysis

Differences in *A*, length *L*, *W*, and the ratio of width to length (*W*/*L*, RWL) among the 12 cultivars were tested using one-way analysis of variance (ANOVA), with cultivar treated as a fixed factor. Tukey’s honestly significant difference (HSD) test was subsequently used for pairwise comparisons among cultivars at the 0.05 significance level [38]. The coefficient of variation (CV = standard deviation/mean × 100%) was calculated to assess intra-cultivar variation for each trait.

To normalize *A*, the two sides of the Montgomery equation (ME) were log-transformed as follows. Here and throughout, log denotes the natural logarithm.

(1) $$A=kLW\;\Leftrightarrow \;\log\left(A\right)=a+\log(LW).$$

In this equation, the intercept *a* is a constant to be estimated. It is apparent that *a* = log(*k*). Unlike the ME, Equation (2) below allows departures from the proportional relationship between *A* and *LW* and is referred to as the power-law equation (PLE). We also took the log-transformation for each side of the PLE:

(2) $$A\;=\;\beta {\left(\mathrm{LW}\right)}^{\alpha }\;\Leftrightarrow \;\log(A)\;=\;\gamma \;+\;\alpha \;\log\;(\mathrm{LW}),$$

where γ = log (β) is a constant to be estimated, and α is the scaling exponent estimated from the data.

According to Thompson’s principle of similarity [12], the two-dimensional area of an object is proportional to its length squared, which is referred to as the square-law equation (SLE) hereinafter. The SLE and its log transformation take the following form:

(3) $$A=cL^{2}\;\Leftrightarrow \;\log\left(A\right)=d+2\log(L),$$

where *d* = log(*c*) is a constant to be estimated.

To fit Equations (1)–(3), reduced major axis (RMA) regression protocols [13,39] were used, as RMA is appropriate for fitting bivariate scaling relationships when neither variable is assumed to be measured without error. To evaluate the goodness of fit for each linear fit, the root-mean-square error (RMSE) was calculated for each of Equations (1)–(3):

(4) $$\mathrm{RMSE}\;=\sqrt{\frac{\sum_{i=1}^{n}{\left(y_{i}-{\hat{y}}_{i}\right)}^{2}}{2\left|\hat{\alpha }\right|n}},$$

where $y_{i}$ is the logarithm of *A*; ${\hat{y}}_{i}$ is its predicted value; $\hat{\alpha }$ is the estimated slope; and *n* is the sample size. Note that the smaller the RMSE, the better the goodness of fit. The coefficient of determination (*r*^2^) was also calculated for Equations (1)–(3) to assess the goodness of fit for each linear fit:

(5) $$r^{2}=1-\frac{\sum_{i=1}^{n}{\left(y_{i}-{\hat{y}}_{i}\right)}^{2}}{\left|\hat{\alpha }\right|\sum_{i=1}^{n}\left(\left|x_{i}-\bar{x}\right|\cdot \left|y_{i}-\bar{y}\right|\right)},$$

where $\bar{x}$ and $\bar{y}$ represent the means of the *x* and *y* values, respectively.

In addition, the percentage error (PE, expressed in %) was used to quantify the relative difference in RMSE between the fitted equations:

(6) $${PE}_{i,j}=\frac{{RMSE}_{i}-{\mathrm{RMSE}}_{j}}{{RMSE}_{i}}\times 100\%,$$

where *i* = 1, 2, and *j* = *i* + 1, representing Equations (1)–(3), respectively. Because Equation (2) includes an additional freely estimated exponent relative to Equation (1), PE_12_ was further used to evaluate the improvement in goodness of fit associated with this additional parameter. Following previous studies, a PE_12_ of 5% was used as a practical reference for interpreting the magnitude of this improvement [21,40].

To quantify uncertainty in the estimated parameters, 95% confidence intervals were calculated using the bootstrap percentile method with 3000 resamples [41,42]. Paired observations were resampled with replacement, and confidence intervals were obtained for the estimated intercepts of Equations (1) and (3) and for the estimated slope of Equation (2); that of $\hat{k}$ was derived from the estimated intercept $\hat{a}$ using $\hat{k}=exp\left(\hat{a}\right)$.

In addition, Pearson’s product–moment correlation coefficient and the *t*-statistic adhering to a *t*-distribution with *n* − 2 degrees of freedom [43] were used to test the correlation between the CV in the ratio of leaf width to length and the RMSE of fitting the SLE at the 0.05 significance level.

All analyses were conducted using R software (version 4.3.1) [37]. The raw data of leaf size measures are accessible online from Table S1.

## 3. Results

**Figure 3:**
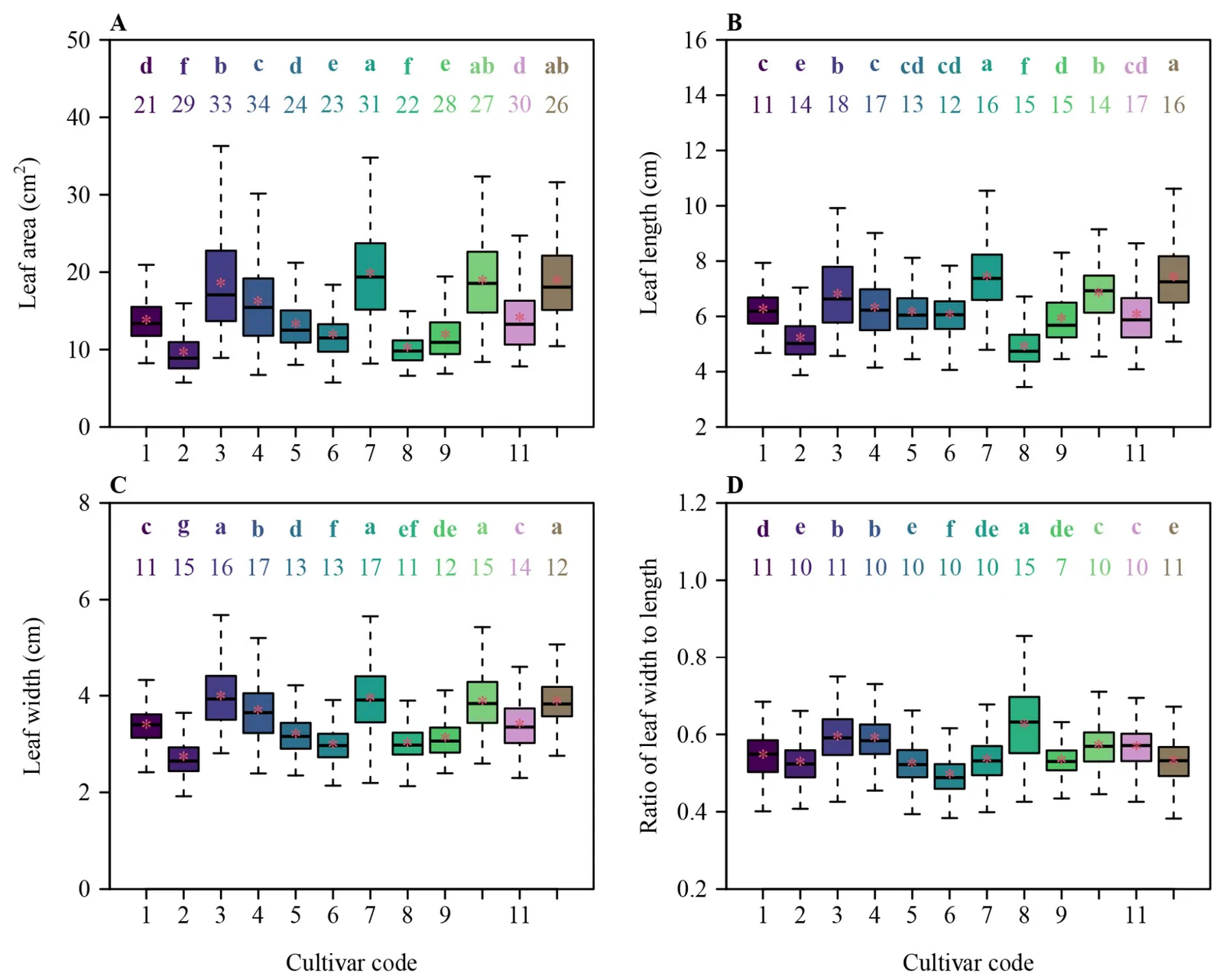
Comparison of individual leaf area (*A*), length (*L*), and width (*W*), and the ratio of leaf width to length (*W*/*L*, RWL) of the 12 *Malus* cultivars. Boxplots of (**A**) leaf areas, (**B**) leaf lengths, (**C**) leaf widths, and (**D**) ratios of leaf width to length across the 12 *Malus* cultivars. The different lowercase letters at the top of boxes indicate a significant difference between cultivars based on the HSD test (α = 0.05); the colored numbers below the letters are the coefficient of variation (CV in %) for each cultivar; the horizontal solid line in each box represents the median; the asterisk represents the mean. The cultivar codes from 1 to 12 represent *M*. ‘Abundance’, *M*. ‘Candymint’, *M*. ‘Cardinal’, *M*. ‘Firebird’, *M*. ‘Indian Summer’, *M*. ‘Louisa Contort’, *M*. ‘Makamik’, *M*. ‘Mary Potter’, *M*. ‘Pink Spires’, *M*. ‘Red Sentinel’, *M*. ‘Snowdrift’, and *M*. ‘Spring Snow’, respectively (see Figure 1 for details).

There were significant differences (*p* < 0.05) in leaf area, length, width, and the ratio of width to length (RWL) among the 12 *Malus* cultivars. For leaf area, cultivar 7 had the largest mean (19.81 cm^2^) and belonged to the highest significance group, together with cultivars 10 and 12, which did not differ significantly from cultivar 7 (Figure 3A). The smallest mean leaf area was observed in cultivar 2 (9.61 cm^2^). For leaf length, cultivars 7 and 12 exhibited the greatest means (7.43 and 7.39 cm, respectively), whereas cultivar 8 had the smallest mean (4.89 cm) (Figure 3B). In terms of leaf width, cultivars 3, 10, 12, and 7 formed the largest group, with cultivar 3 showing the highest numerical mean (3.99 cm), and cultivar 2 was the narrowest (2.72 cm) (Figure 3C). For RWL, cultivar 8 had the largest mean (0.626), whereas cultivar 6 had the smallest (0.494) (Figure 3D).

**Figure 4:**
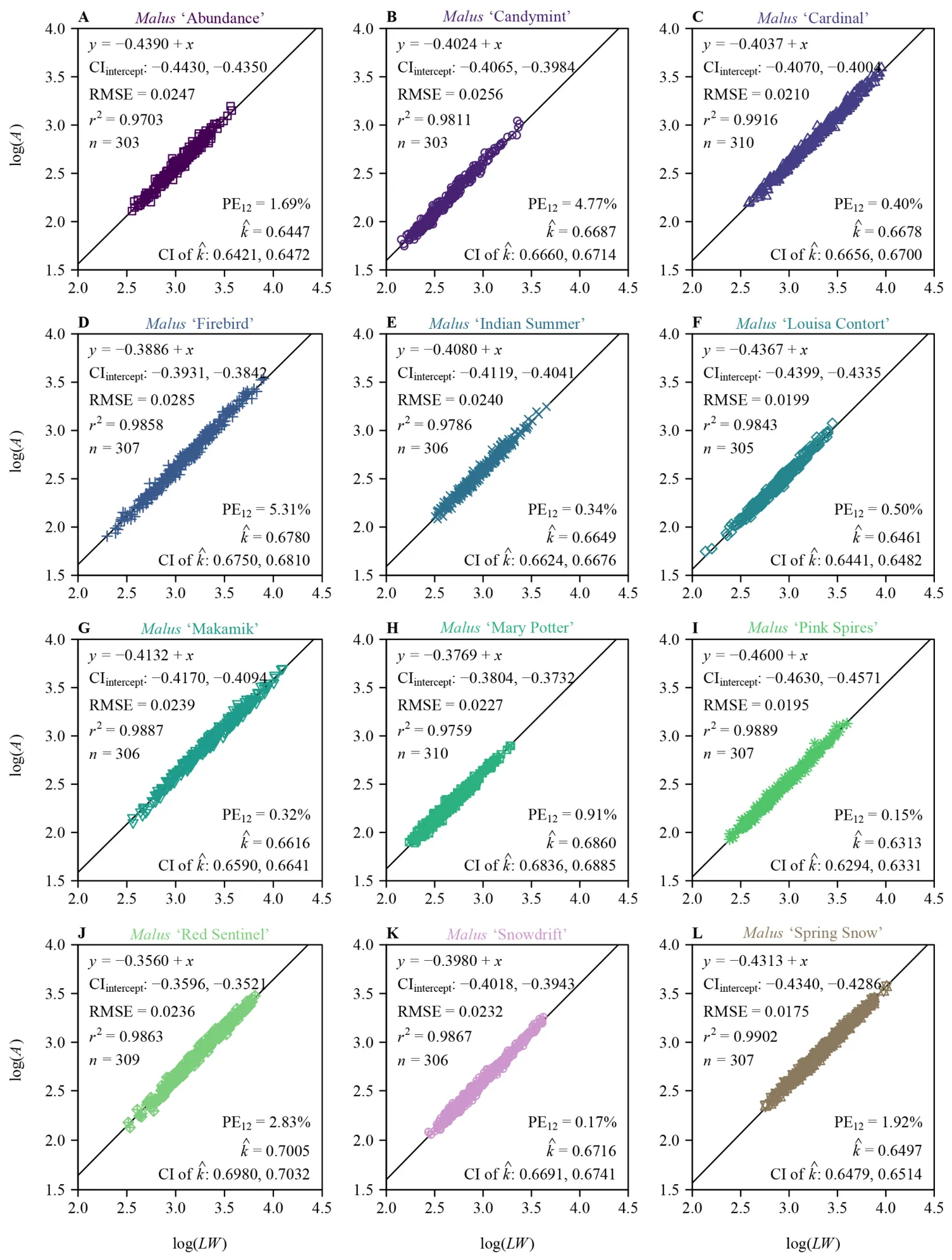
Bivariate relationship between leaf area (*A*) and the product of leaf length and width (*LW*) on a log–log axis of the 12 *Malus* cultivars. The icons represent the observations; CI_intercept_ represents the 95% confidence interval of the estimated intercept; RMSE is the root-mean-square error of linear fitting; *r*^2^ is the coefficient of determination for linear fitting; and *n* is the sample size, i.e., the total number of leaves measured for each cultivar. PE_12_ represents the percent error between the Montgomery equation (i.e., Equation (1)) and the power-law equation (i.e., Equation (2)). $\hat{k}$ represents the estimated proportionality coefficient of the ME, and CI of $\hat{k}$ represents the 95% confidence interval of the estimated Montgomery parameter. Panels (**A**–**L**) represent the 12 *Malus* cultivars, respectively, with different icons indicating different cultivars.

**Figure 5:**
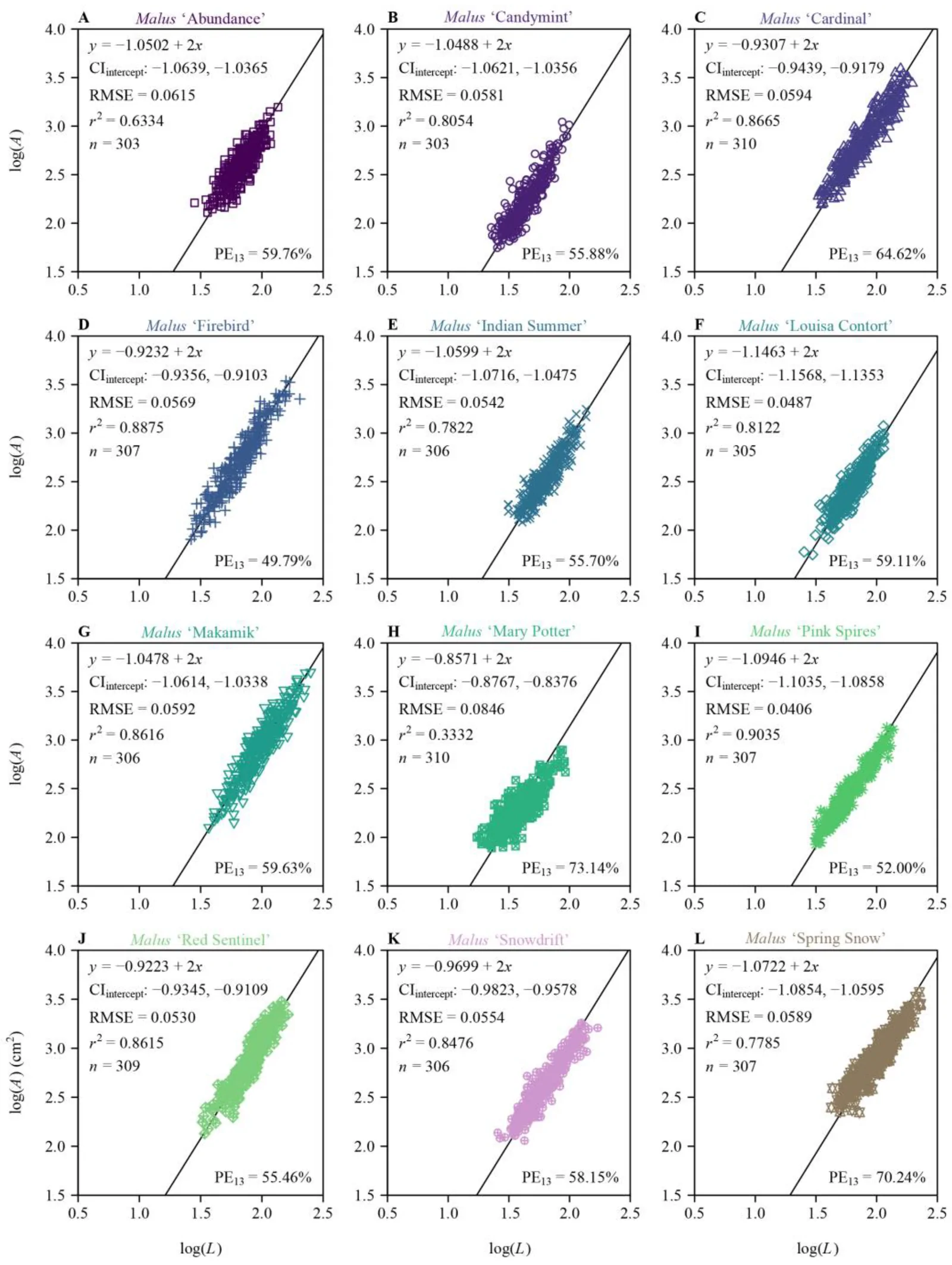
Bivariate relationship between leaf area (*A*) and the square of leaf length (*L*) on a log–log axis of the 12 *Malus* cultivars. The icons represent the observations; CI_intercept_ represents the 95% confidence interval of the estimated intercept; RMSE is the root-mean-square error of linear fitting; *r*^2^ is the coefficient of determination for linear fitting; and *n* is the sample size, i.e., the total number of leaves measured for each cultivar. PE_13_ represents the percent error between the Montgomery equation (i.e., Equation (1)) and the square-law equation (i.e., Equation (3)). Panels (**A**–**L**) represent the 12 *Malus* cultivars, respectively, with different icons indicating different cultivars.

The Montgomery equation provided a good fit for each of the 12 cultivars (*r*^2^ ≥ 0.9703 and RMSE ≤ 0.0285; Figure 4). The scaling exponents estimated from Equation (2) were consistently close to unity across the 12 cultivars ($\hat{\alpha }$ = 0.9795–1.0439; Table S2). The 95% confidence intervals included unity for six cultivars and excluded unity for the other six, indicating that small but statistically detectable departures from strict isometry occurred in some cultivars. Nevertheless, the percentage error between Equations (1) and (2) (PE_12_) was below the 5% reference value for 11 of the 12 cultivars, while *Malus* ‘Firebird’ (cultivar 4) had a PE_12_ of 5.31% (Figure 4), indicating that allowing the additional exponent generally resulted in only a small reduction in RMSE. In contrast, the square-law equation (SLE) provided an inferior fit for each of these cultivars (0.3332 ≤ *r*^2^ ≤ 0.9035 and RMSE > 0.0406) compared to the ME, with the percentage error between Equations (1) and (3) (PE_13_) ≥ 49% for all cultivars (Figure 5).

**Figure 6:**
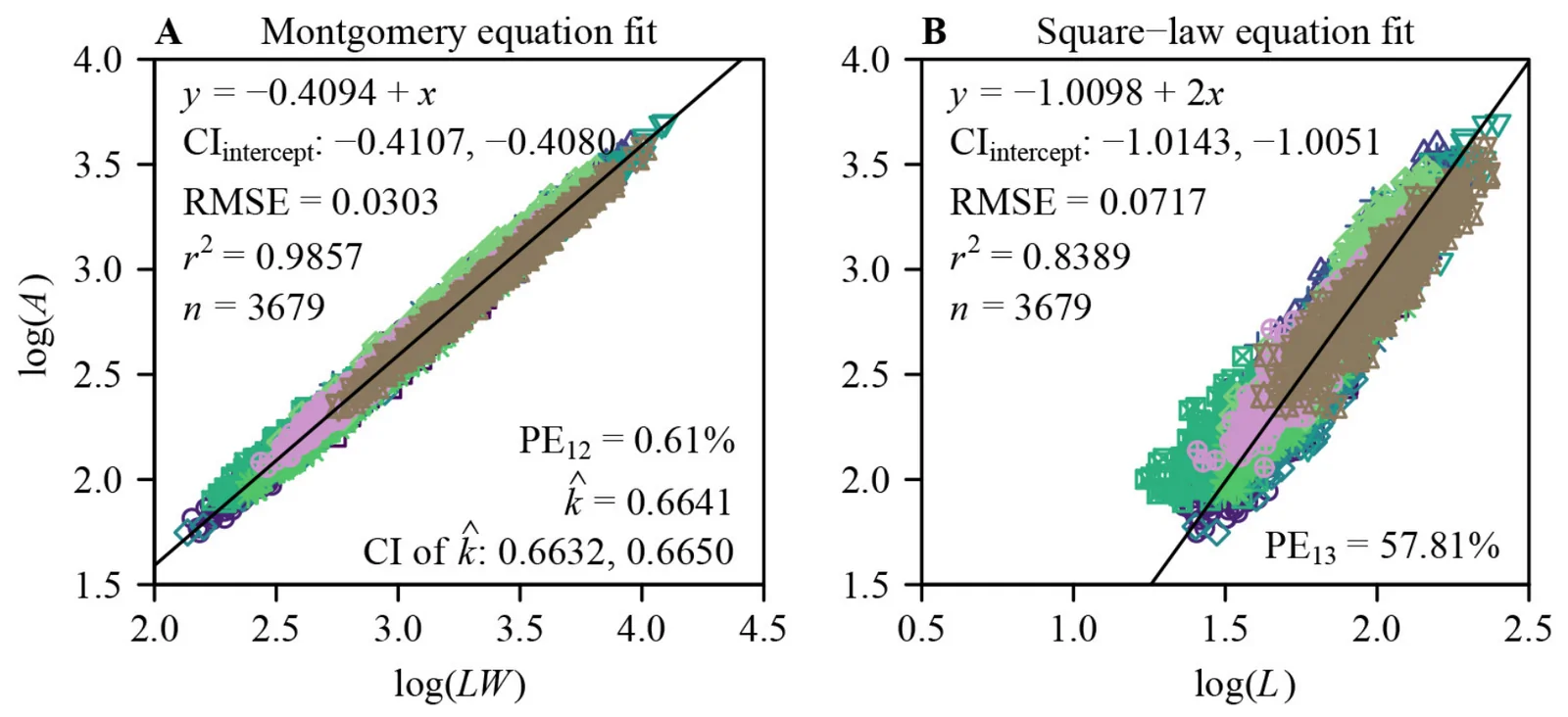
Bivariate relationship (**A**) between individual leaf area (*A*) and the product of leaf length and width (*LW*), and (**B**) between *A* and *L* on a log–log scale for the pooled data of the 12 *Malus* cultivars. The icons represent the observations; CI_intercept_ represents the 95% confidence interval of the estimated intercept; RMSE is the root-mean-square error of linear fitting; *r*^2^ is the coefficient of determination for linear fitting; and *n* is the sample size, i.e., the total number of leaves measured for the 12 cultivars. PE_12_ represents the percent error between the Montgomery equation (i.e., Equation (1)) and the power-law equation (i.e., Equation (2)). $\hat{k}$ represents the estimated proportionality coefficient of the ME, and CI of $\hat{k}$ represents the 95% confidence interval of the estimated Montgomery parameter. PE_13_ represents the percent error between the Montgomery equation (i.e., Equation (1)) and the square-law equation (i.e., Equation (3)). Different icons represent different cultivars, consistent with Figure 4 and Figure 5.

**Figure 7:**
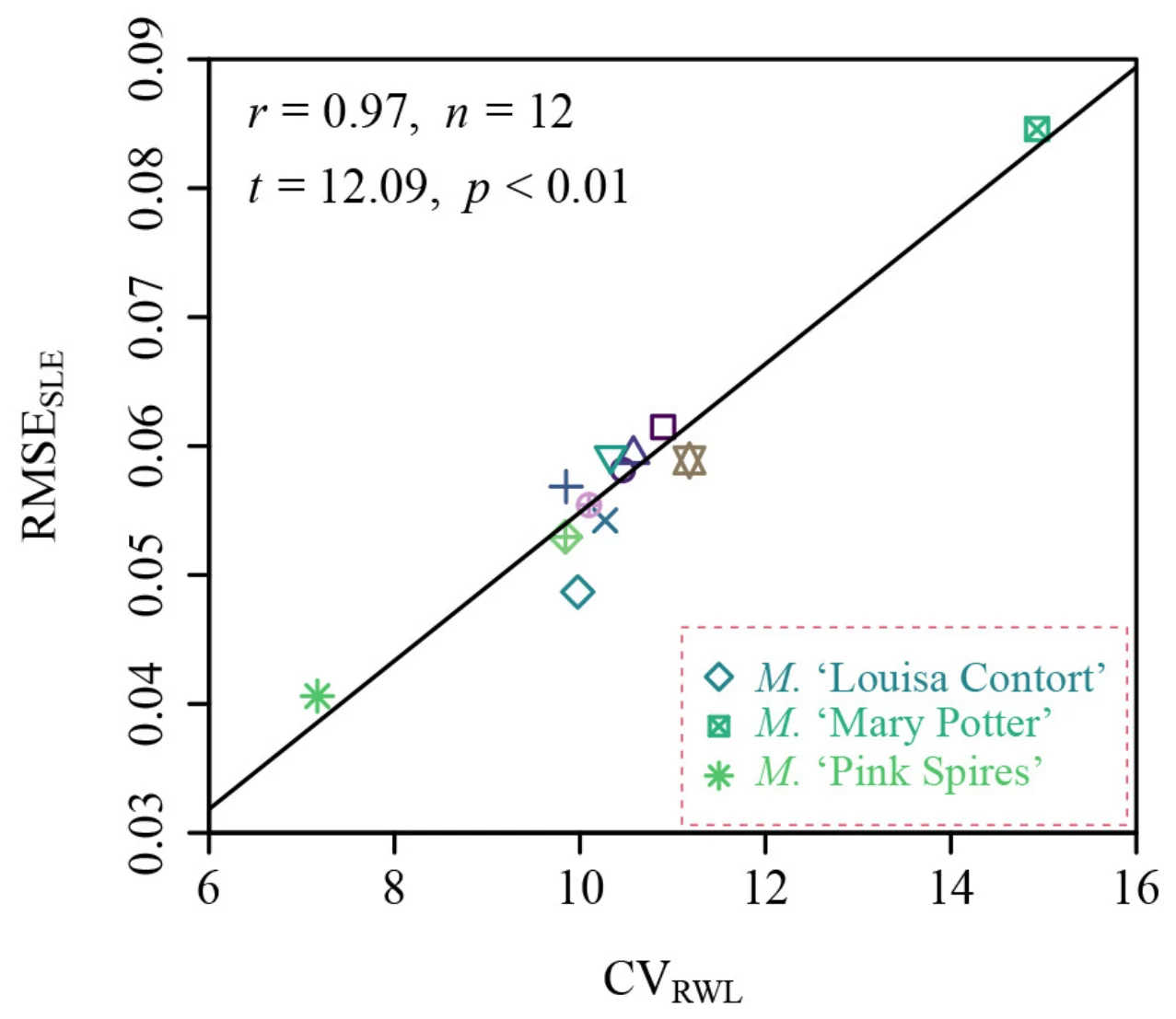
Correlation between the coefficient of variation (CV) in the ratio of leaf width to length (RWL) (CV_RWL_) and the root-mean-square error (RMSE) of fitting the square-law equation (SLE, i.e., the hypothesis that *A* ∝ *L*^2^) (RMSE_SLE_). Pearson’s product–moment correlation coefficient and the *t*-statistic following a *t* distribution with *n* − 2 degrees of freedom were used to test the correlation between the CV_RWL_ and the RMSE_SLE_ at the 0.05 significance level. *r* is the correlation coefficient; *n* is the sample size. Different icons represent different cultivars, consistent with Figure 4, Figure 5 and Figure 6.

For the pooled data of 3679 leaves, the ME also provided a good fit (*r*^2^ = 0.9857 and RMSE = 0.0303; Figure 6A), and the estimated Montgomery parameter ($\hat{k}$) was equal to 0.6641 with 95% confidence intervals (CIs) of 0.6632 and 0.6650. In comparison, the SLE produced a lower *r*^2^ of 0.8389 and a higher RMSE of 0.0717 for the same pooled dataset (Figure 6B). Furthermore, the PE_12_ for the pooled data was 0.61%, whereas PE_13_ was 57.81% (Figure 6B). Across the 12 cultivars, the coefficient of variation in the ratio of leaf width to length (CV_RWL_) ranged from 7.1% to 14.9%, whereas RMSE_SLE_ ranged from 0.0406 to 0.0846 (Figure 7). A strong positive correlation was detected between CV_RWL_ and RMSE_SLE_ (*r* = 0.97, *p* < 0.01), indicating that greater within-cultivar variation in leaf shape was associated with poorer goodness of fit of the SLE.

## 4. Discussion

Leaf size and shape are critical functional traits that directly influence photosynthetic capacity, water-use efficiency, competitive ability, and plant responses to environmental gradients [1,3,44]. Because leaf area is closely related to leaf geometry, simple linear dimensions such as leaf length (*L*) and width (*W*) have long been used to develop non-destructive approaches for estimating leaf area (*A*). However, the goodness of fit of these relationships may vary with leaf morphology, even among closely related cultivars.

The 12 *Malus* cultivars examined here have simple leaves ranging from ovate to elongated elliptic, with varying degrees of marginal serration and lobing (Figure 1). Such morphological variation provides an opportunity to evaluate geometric leaf-area relationships within a single genus. The following sections discuss three key aspects arising from our morphometric and scaling analyses. First, we evaluated the applicability of the Montgomery equation (ME) across diverse *Malus* cultivars and compared our findings with previous studies on other plant groups. Second, we examined the numerical range of the Montgomery parameter (*k*) and its biological significance. Third, we compared the ME with the square-law equation (SLE), with particular attention to the association between variation in the ratio of leaf width to length (RWL) and SLE performance.

### 4.1. Applicability of the Montgomery Equation

The ME was originally developed for linear, strap-shaped corn leaves [16]. It has since been validated for diverse leaf shapes, including elliptical, orbicular, ovate, hastate, triangular, deltoid, fan-shaped, and lobed forms. Its applicability has also been confirmed across different life forms, including herbs, woody plants, and vines [18,20,21,22,23,24,25]. It is now regarded as a reliable, non-destructive method for leaf-area estimation for broad-leaved plants. The present study further evaluated the applicability of the ME for estimating individual leaf area across 12 *Malus* cultivars.

The ME provided a good fit for all 12 *Malus* cultivars (*r*^2^ ≥ 0.9703 and RMSE ≤ 0.0285; Figure 4). The scaling exponents estimated from the more general power-law equation (PLE) remained close to unity ($\hat{\alpha }$= 0.9795–1.0439; Table S2), although the 95% confidence intervals did not include unity for all cultivars. Thus, small but statistically detectable departures from strict isometry occurred in some cultivars. Nevertheless, the percentage error between Equations (1) and (2) (PE_12_) was below the 5% reference value for 11 of the 12 cultivars, while *Malus* ‘Firebird’ had a PE_12_ of 5.31% (Figure 4), indicating that allowing the scaling exponent to vary generally resulted in only limited improvement in goodness of fit over the proportional ME. Taken together, the low RMSE values and the generally small PE_12_ values support the use of the ME as a simple description of the relationship between leaf area and the product of leaf length and width in the investigated *Malus* cultivars.

### 4.2. Numerical Range and Geometric Interpretation of the Montgomery Parameter

The estimated Montgomery parameter ($\hat{k}$) for the pooled dataset was 0.6641 (95% CI: 0.6632, 0.6650; Figure 6A), representing the overall relationship across the combined sample. Because the ME is expressed as *A* = *kLW*, *k* represents the ratio of actual leaf area to the area of the rectangle defined by leaf length and maximum width, i.e., *A*/(*LW*), and thus reflects overall leaf geometry. This value falls within the theoretical range of 1/2 to π/4 that has been proposed for other broad-leaved plants [25]. Geometrically, an idealized triangular leaf would yield *k* = 1/2, whereas an elliptical leaf would yield *k* = π/4 [21,25]. These reference values provide a geometric perspective for interpreting the magnitude of *k* and support its use as a concise quantitative descriptor of overall leaf geometry. The *k* values obtained for the 12 *Malus* cultivars were also broadly similar to those reported for 12 Rosaceae species by Shi et al. [20]. In addition, the pooled RMSE in the present study (RMSE = 0.0303; Figure 6A) was lower than the value reported by Shi et al. [20] for the Rosaceae dataset (RMSE = 0.0686). Although these values were obtained from different datasets, the comparison suggests that the ME can maintain a high goodness of fit when applied within a narrower taxonomic group.

Although the ME holds for all cultivars, the estimated *k* values among the 12 cultivars varied from 0.6313 to 0.7005 (Figure 4). This variation may reflect cultivar-level differences in leaf geometry and highlights the potential value of cultivar-specific *k* values when greater estimation accuracy is required. Nevertheless, inferential comparisons among cultivars should be interpreted with appropriate caution because multiple leaves were sampled from individual trees, whereas the source-tree identity of each leaf was not retained. Consequently, potential within-tree dependence could not be explicitly accounted for in the analyses. Future studies retaining tree identity would allow this hierarchical sampling structure to be incorporated directly.

### 4.3. Influence of the Coefficient of Variation in the Ratio of Leaf Width to Length on the Effectiveness of Thompson’s Square-Law Equation

The SLE has long been used as a convenient approximation for estimating area from a single linear dimension [12]. In the present study, however, the ME exhibited a better fit than the SLE for each of the 12 cultivars (Figure 5), and the percentage error between Equations (1) and (3) was at least 49% for all cultivars and for the pooled data (PE_13_ ≥ 49%). Residual diagnostics further supported this difference in fit, with the SLE showing greater residual dispersion and more pronounced negative trends across several cultivars than the ME and PLE (Figures S1–S3). These patterns highlight the limitations of relying solely on leaf length. To explore the basis of these large PE_13_ values, we further examined whether the goodness of fit of the SLE was associated with variation in leaf shape. Our data showed that the goodness of fit of this simplified relationship decreased as leaf-shape variation increased. The RMSE of the SLE increased from 0.0406 to 0.0846 as the CV in RWL increased from 7.1% to 14.9%, and the two variables were strongly positively correlated (*r* = 0.97, 95% CI: 0.8848, 0.9911, *p* < 0.01; Figure 7). This positive correlation is consistent with the geometric expectation proposed by Shi et al. [20], suggesting that greater variation in RWL is associated with poorer goodness of fit of the SLE. Recently, Qiu et al. [45] reported a nearly identical relationship in a single bamboo species (*Semiarundinaria densiflora*), where the RMSE of the SLE increased significantly with increasing CV of RWL across 121 culms.

The present results extend previous observations of the relationship between leaf-shape variation and SLE goodness of fit to cultivar-level comparisons within *Malus*. The consistently large PE_13_ values, together with the positive relationship between CV in RWL and RMSE of the SLE, indicate that incorporating both leaf length and width provides a substantially better fit than relying on leaf length alone for the *Malus* cultivars examined here. In addition, the ME provides a biologically meaningful parameter (*k*) that quantitatively reflects leaf geometry. However, several questions remain unresolved. First, it remains unclear whether the estimated *k* values remain stable across different leaf developmental stages, growing seasons, and environmental conditions. Second, RWL captures only one aspect of leaf shape, and the extent to which other geometric features, such as marginal lobing or asymmetry, influence the performance of the ME and SLE remains to be tested. Future studies could explore direct geometric fitting of leaf outlines to better quantify leaf-shape variation beyond linear dimensions, similar to modeling fruit 2-D profiles [46]. Third, the relationships observed here should be further examined across a broader range of plant taxa with diverse leaf morphologies. Future studies addressing these questions would help establish the generality and practical applicability of these leaf-area relationships across different plant groups and environmental conditions.

## 5. Conclusions

The present study demonstrated that the Montgomery equation (ME) provides a consistently good fit to the relationship between leaf area and the product of leaf length and width across the examined *Malus* cultivars. The estimated Montgomery parameter fell within the established geometric reference range, while its variation among cultivars reflected differences in the proportional relationship between leaf area and the product of leaf length and width. Compared with the square-law equation (SLE), the ME consistently exhibited better goodness of fit, highlighting the advantage of incorporating both leaf length and width in leaf-area estimation. Furthermore, the strong positive correlation between variation in the ratio of leaf width to length and the RMSE of the SLE suggests that greater within-cultivar leaf-shape variation is associated with poorer goodness of fit of the length-only relationship. Collectively, these findings support the practical application of the ME as a simple, non-destructive approach for leaf-area estimation in *Malus*, providing a useful basis for physiological, ecological, and horticultural research.

## Data Availability

The data can be found in the online Supplementary Table S1.

## Supplementary Materials

The following supporting information can be downloaded at https://www.mdpi.com/article/10.3390/plants15213359/s1, Table S1: Leaf length, width, and area data of 12 *Malus* cultivars; Table S2: Scaling exponent for the power-law equation (PLE) across 12 *Malus* cultivars; Figure S1: Residual diagnostics for the Montgomery equation (ME) across 12 *Malus* cultivars; Figure S2: Residual diagnostics for the power-law equation (PLE) across 12 *Malus* cultivars; Figure S3: Residual diagnostics for the square-law equation (SLE) across 12 *Malus* cultivars.
